# Preparation and Characterization of Microcrystalline Wax/Epoxy Resin Microcapsules for Self-Healing of Cementitious Materials

**DOI:** 10.3390/ma14071725

**Published:** 2021-03-31

**Authors:** Wei Du, Quantao Liu, Runsheng Lin, Xin Su

**Affiliations:** 1 School of Material Science and Chemical Engineering, Ningbo University, Ningbo 315211, China; 2 State Key Laboratory of Silicate Materials for Architectures, Wuhan University of Technology, Wuhan 430070, China; liuqt@whut.edu.cn; 3 College of Engineering, Department of Architectural Engineering, Kangwon National University, Chuncheon-si 200-701, Korea; linrunsheng@kangwon.ac.kr

**Keywords:** cementitious materials, microcapsules, melt–dispersion–condensation method, microcrystalline wax, self-healing

## Abstract

Self-healing of cracks in cementitious materials using healing agents encapsulated in microcapsules is an intelligent and effective method. In this study, microcapsules were prepared by the melt–dispersion–condensation method using microcrystalline wax as the shell and E-51 epoxy resin as the healing agent. The effects of preparation process parameters and microcrystalline wax/E-51 epoxy resin weight ratio on the core content, particle size distribution, thermal properties, morphology, and chemical composition of microcapsules were investigated. The results indicated that the optimal parameters of the microcapsule were microcrystalline wax/E-51 epoxy resin weight ratio of 1:1.2, stirring speed of 900 rpm, and preparation temperature of 105 °C. The effects of microcapsules on pore size distribution, pore structure, mechanical properties, permeability, and ultrasonic amplitude of mortar were determined, and the self-healing ability of mortar with different contents of microcapsules was evaluated. The optimal content of microcapsules in mortars was 4% of the cement weight, and the surface cracks of mortar containing microcapsules with an initial width of 0.28 mm were self-healed within three days, indicating that microcapsules have excellent self-healing ability for cementitious materials.

## 1. Introduction

Cementitious materials are widely used in building construction materials and have excellent mechanical properties and workability [1]. However, the formation of cracks in cementitious materials is inevitable due to their internal porosity and exposure to complex service environments [2,3,4]. Cracks provide a direct route for the penetration of harmful substances (chloride ions, sulfate ions, etc.) into the concrete [5], leading to corrosion of reinforcement [6,7] and even complete failure of the concrete structure. In order to ensure the safety of the building structure and prolong its service life, the building must be extensively overhauled or planned maintenance during the course of its use. At present, the monitoring and maintenance of concrete relies on manual intervention. However, current manual inspection techniques make it difficult to locate internal cracks in buildings and to detect and repair internal microcracks in a timely manner, which can also incur high maintenance costs [8,9]. Therefore, it is imperative to develop new materials with low cost and high performance, especially for cementitious materials with self-healing ability, which can effectively reduce maintenance, extend service life, and improve safety [10,11].

Scientists have proposed the concept of self-healing of cementitious materials through imitating the self-healing function of biological injuries [12]. The organisms have regenerative functions and can repair wounds on their own after matrix injury. After an animal fracture, blood forms a clot that initially connects the fracture, and then osteoblasts form new bone tissue that transforms into normal bone and heals the wound. The repair component of self-healing concrete mimics the action of animal blood. After cracks appear in the matrix, the cracks reach the damaged surface and are filled by physical or chemical action to heal automatically, thus restoring the properties of the material.

The concrete self-healing techniques mainly include the crystalline admixtures method, microbial method, and microencapsulation method. The crystalline admixtures could promote the un-hydrated cement to form the crystalline product in concrete, and thus could be applied to heal the cracks [13]. However, this self-healing method can only be performed in the presence of water and has a relatively long healing cycle [14,15]. The microbial method is used to heal cracks by inducing direct or indirect production of calcium carbonate from metabolites through the metabolism of bacterial spores [16]. Although the microbial method has been able to heal cracks in the laboratory, the presence of bacterial spores reduces the strength of the cement matrix and makes it difficult to meet the requirements of important engineering applications [17,18]. Compared with crystalline admixtures and microbial methods, microcapsule-based self-healing is considered the most promising method for repairing cracks in cementitious materials because of its good self-healing ability and low cost [19].

As a container, the microcapsules store the healing agent in advance until cracks form in the cementitious material. At that time, the microcapsules will rupture and release the healing agent, allowing the cracks to self-heal. The key issues of the microencapsulation method include the following: (1) the healing agent must be able to encapsulate into the microcapsules; (2) the microcapsules need to form a good bond with the cementitious material, and their morphology and shell roughness should meet certain requirements; (3) they will not be destroyed during mixing and can be stored stably in the cementitious material. In addition, the selections of core and shell materials also affect the self-healing ability of microcapsules. The shell material needs to have a certain strength that can be well preserved during the specimen preparation. However, if the strength is too high, the microcapsules cannot break in time when cracks appear. The choice of the core material should take into account not only the fluidity of the healing agent, but also the actual healing effect that can be achieved by the microcapsules. Therefore, a shell material with certain strength and a core material with good fluidity and remarkable healing effect must be selected to prepare microcapsules for better self-healing of cracks in cementitious materials.

Many reviews of literature on self-healing materials have shown that dilute epoxy resins as healing agents have good self-healing ability for cementitious materials [20,21,22,23]. At present, the shells of microcapsules are usually chemically synthesized from thermosetting resins (polyurethane, urea-formaldehyde, melamine-formaldehyde, etc.) by in-situ polymerization or interfacial polymerization [24,25,26]. These microcapsules easily maintain integrity during concrete mixing and forming (the shell material has high strength), but they also increase the difficulty of breaking and releasing the healing agent under stress [27,28]. In order to solve this problem, low strength thermoplastic materials, such as microcrystalline wax, can be selected as shell materials of microcapsules, which can make microcapsules easy to rupture and release the healing agent under external forces.

In this paper, E-51 epoxy resin was encapsulated into microcrystalline wax to prepare microcapsules using the melt–dispersion–condensation method. The effects of microcrystalline wax/epoxy resin weight ratio, stirring speed, and preparation temperature on the core content of microcapsules were studied. The particle size distribution, thermal properties, morphology, and chemical composition of the microcapsules were characterized. The flexural strength, compressive strength, pore size distribution, chloride diffusion coefficient, and amplitude of mortars were determined, and the self-healing ability of mortars with different contents of microcapsules was evaluated. The mortars containing microcapsules were tested for crack width after three days of self-healing in the laboratory.

## 2. Materials and Methods

### 2.1. Materials

**Figure 1:**
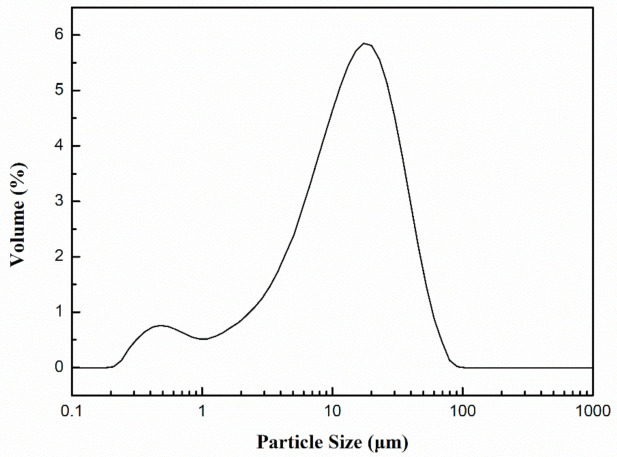
Particle size distribution of cement.

Microcrystalline wax (melting point: 87–92 °C) was obtained from Ningbo Yanyu Biotechnology Co., Ltd., Ningbo, China). E-51 epoxy resin and N, N-dimethylformamide were purchased from Ningbo Yongchuan Biotechnology Co., Ltd., Ningbo, China). 2-ethyl-4-methylimidazole, perfluorotributylamine, and acetone were supplied by Shanghai Molbase Biotechnology Co., Ltd., Shanghai, China). Portland cement (P.O 42.5) was bought from Anhui Conch Cement Co., Ltd., Wuhu, China. The chemical composition of cement is shown in Table 1, and the particle size distribution of cement is described in Figure 1. River sand (modulus: 2.35) was applied by Ningbo Pulunxiang building materials Co., Ltd., Ningbo, China).

### 2.2. Preparation of Microcapsules

**Figure 2:**
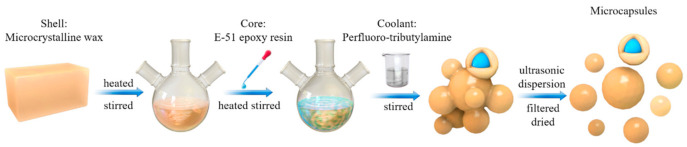
Schematic diagram of the microcapsule preparation process.

Microcapsules were prepared by the melt–dispersion–condensation method. The melt–dispersion–condensation method is a physical preparation method, and no chemical reaction occurs during the preparation of microcapsules, avoiding possible chemical contamination from the source. It is a green preparation method that conforms to the concept of sustainable development. Firstly, the microcrystalline wax was melted and dissolved in a three-necked round-bottom flask to prepare microcapsules at 95, 105, and 115 °C, respectively. Then, E-51 epoxy resin diluted with N, N-dimethylformamide (DMF) was added dropwise to the liquid microcrystalline wax for more than 30 s. The amount of DMF was 15% of the weight of E-51 epoxy resin, and the shell/core weight ratios were 1.2:1, 1:1, 1:1.2, and 1:1.4, respectively. The shell/core mixture were stirred for 4 h at a certain stirring speed (300, 600, 900, and 1200 rpm). Then, perfluorotributylamine was added to the mixture, and the temperature was rapidly reduced to below the melting point of microcrystalline wax to obtain the microcapsule suspension. After that, the suspension was oscillated for 15 min by ultrasonic wave. Finally, the microcapsules were filtered out from the mixture and dried for 24 h at 75 °C. Figure 2 depicts a schematic diagram of the preparation process.

### 2.3. Preparation of Mortars

Two kinds of mortar specimens were prepared separately to test the mechanical properties and impermeability of the mortars. The preparation process of mortar containing microcapsules was as follows: First, cement, sand, and microcapsules were stirred in a mortar mixer for 60 s. Then, water and 2-ethyl-4-methylimidazole were added to the mortar mixer, and stirring was continued for 2 min. The content of 2-ethyl-4-methylimidazole was 20% of the mass of diluted epoxy resin. The prism samples with the casting size of 40 mm × 40 mm × 160 mm were tested for mechanical properties. In the chloride ion diffusion test, cylindrical samples of size ϕ 100 mm× 50 mm were poured. Finally, the mortar was demolded after 24 h and moved to a standard curing room (20 ± 2 °C, relative humidity ≥ 95%) to cure for 28 days. The formulations of mortars are shown in Table 2.

### 2.4. Measurement and Characterization of Microcapsules

#### 2.4.1. Core Content

In order to determine the core content of the microcapsules, a certain mass of microcapsules was first weighed and then thoroughly ground so that all the epoxy resin flowed out. Then, the residual microcrystalline wax was soaked in acetone for 24 h. Finally, the residual microcrystalline wax was dried and weighed [29]. The core content of microcapsules was computed by Equation (1).

(1) $$\varepsilon =\frac{m_{0}-m_{1}}{m_{0}}\times 100\%$$

where ε is the microcapsule core content (%), *m*_0_ is the microcapsule mass (g), and *m*_1_ is the residual microcrystalline wax mass (g).

#### 2.4.2. Size Distribution

The particle size distribution of microcapsules was determined using a laser particle size analyzer (Mastersizer 2000, Malvern Instruments Ltd., Malvern, UK) [30]. The specific testing process can be found in the Supporting Information.

#### 2.4.3. Thermal Property

The thermal properties of microcapsules were characterized using a thermal analyzer (STA449F3, NETZSCH Group, Selb, Germany) under a nitrogen atmosphere (20 mL/min) in the temperature range 30–700 °C and at a heating/cooling rate of 10 °C/min, which was adopted in another reference [31]. At first, the temperature and heat flow calibrations were done using indium under nitrogen atmosphere. A known weight of sample was placed in a sealed aluminum pan for measurement, while an empty pan was used for reference. The thermal properties and thermal stability were determined using the Universal Analysis 2000 TA software package (TA Instruments, New Castle, DE, USA).

#### 2.4.4. Morphology

The morphology of the microcapsules was observed by scanning electron microscopy (SEM) (S-4800, Hitachi, Tokyo, Japan). To observe the thickness of the microcapsule shell, the microcapsules were cut through a sharp blade, cleaned with acetone, and dried before SEM observation. A gold film was sprayed on the surface of intact and broken microcapsules, and a voltage of 3 kV was applied. The test procedure of SEM was carried out according to the methods in other reference [32].

#### 2.4.5. FTIR Spectrum

Microcrystalline wax, E-51 epoxy resin, N, N-dimethylformamide, and microcapsules were characterized through Fourier transform infrared spectroscopy (Nexus, Thermo Nicolet Corporation, Madison, WI, USA). The microcrystalline wax and microcapsules were compressed into thin sheets using potassium bromide. E-51 epoxy resin and N, N-dimethylformamide were brushed on the potassium bromide sheet. The test conditions were set as follows: 64 scans, 4 cm^−1^ resolution, and 4000–400 cm^−1^ test range. The test procedure for FTIR was performed by referring to methods in other reference [27].

### 2.5. Evaluation of Self-Healing Ability of Pre-Load Mortars

#### 2.5.1. Mechanical Property

After standard curing for 28 days, the flexural strength and compressive strength of the mortar were determined and expressed as f_a0_ and f_b0_, respectively. The testing method was based on a standard [33]. Three specimens were taken from each group to calculate the average flexural strength, and six specimens were taken for the mortar compressive strength test. To obtain pre-damaged mortar specimens, the mortar was loaded to 60%f_b0_. The pre-damaged mortar specimens were left in the laboratory (20 °C, 50% RH) for three, seven, and 14 days for self-healing. Finally, the specimens were reloaded, and the compressive strength recovery ratio of the mortar was measured according to Equation (2).

(2) $$\delta =\frac{f_{\mathrm{bt}}}{f_{b0}}\times 100\%$$

where δ is the compressive strength recovery ratio of mortar, f_b0_ is the compressive strength of mortar after 28 days of standard curing, and f_bt_ is the compressive strength of mortar after self-healing.

#### 2.5.2. Pore Size Distribution

A nuclear magnetic resonance spectrometer (MesoMR25, Suzhou Newman Analytical Instrument Co., Ltd., Suzhou, China) was applied to characterize the pore size distributions of mortars [34]. The pore size distributions of mortars after 60%f_b0_ pre-load and self-healing for 14 days were measured. The specific testing process can be found in the Supporting Information. The pore diameter was calculated by Equation (3).

(3) $$\frac{1}{T_{2}}=\rho (\frac{S}{V}{)}_{\mathrm{pore}}$$

where T_2_ is the relaxation time of water in the pore, ρ is the surface relaxation rate (70 μm/ms), and (S/V) _pore_ is the pore surface area to volume ratio.

#### 2.5.3. Impermeability

The initial chloride diffusion coefficients of the specimens were determined according to the Nordic standard NTBUILD 443 [35]. Then all specimens were preloaded at 60%f_b0_ and the chloride diffusion coefficients of the control mortar after 60%f_b0_ preloading were recorded as M-0-60. The pre-damaged specimens were self-healed for three, seven, and 14 days, respectively. The recovery rates were calculated according to Equation (4).

(4) $$\theta =-\frac{\theta_{t}{-\theta }_{0}}{\theta_{0}}\times 100\%$$

where θ is the chloride diffusion coefficient recovery rate (%), θ_0_ is the chloride diffusion coefficient of the control mortar after pre-loading (10^−12^ m^2^/s), and θ_t_ is the chloride diffusion coefficient of the pre-loaded control mortar after self-healing (10^−12^ m^2^/s).

#### 2.5.4. Ultrasonic Testing

For cementitious materials, mechanical properties, permeability, and compactness are important factors that affect their quality. Accidents caused by these factors bring great losses to individuals and society. Many researchers have studied the macroscopic mechanical properties and permeability of concrete. However, these tests are usually destructive to the test object. In practical applications, it is usually necessary to observe and evaluate various properties of buildings without damaging them, so the application of nondestructive testing techniques is of great importance. Ultrasonic testing techniques are the fastest growing and most widely used of all non-destructive testing technologies and have a very important position [36].

Generator (AFG3022C, Tektronix, Shanghai, China) is used to generate and transmit ultrasonic waves. An oscilloscope (MDO 3024, Tektronix, Shanghai, China) was applied to receive and characterize the ultrasonic waves. The ultrasonic frequency was 107 kHz, and the voltage was controlled at ±5 V. The radial piezoelectric ultrasonic transducer emitted ultrasonic waves when collecting data. Standard transducers were attached to both sides of the mortar, and the coupling agent was petroleum jelly. During the test, data and pictures were automatically saved on a computer connected to the transducer. Finally, the ultrasonic signal was converted to the frequency domain from the time domain by the Fast Fourier Transform (FFT) function in the plotting software. Ultrasound can propagate in different media and has a high repeatability of waveforms and frequencies when propagating at a steady state of energy. Therefore, the self-healing ability of mortar could be evaluated by testing these parameters. The mortar was measured after 60%f_b0_ preloading and self-healing for 14 days.

### 2.6. Self-Healing of Surface Cracks

**Figure 3:**
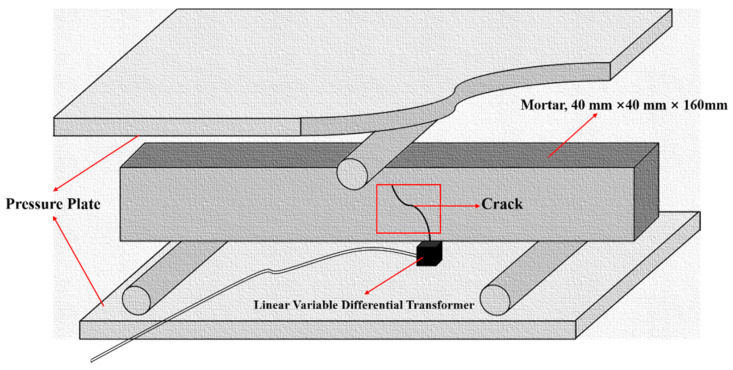
Schematic diagram of pre-cracked mortar by the three-point bending method.

In order to evaluate the self-healing capacity of concrete, after 28 days of standard curing, the mortar specimens were pre-cracked up to different levels of residual crack opening. Specimens were pre-cracked employing the three-point bending method; the deformation of the specimen was measured using two Linearly Varying Differential Transformers (LVDT), which are fixed in the middle of the bottom of the specimen, as shown in Figure 3. The bending strain at the bottom of the specimen was measured from the LVDT as a measure of the crack opening width. The average signal of the two LDVTs was used as feedback information to control the deformation in the test, and the loading was stopped when the predetermined crack width was reached [37,38]. The crack widths of mortars containing microcapsules before and after three days of self-healing in the laboratory were determined by a crack tester (PES-30, Botte Instrument Co., Ltd., Wuhan, China).

## 3. Results and Discussion

### 3.1. Core Content

**Figure 4:**
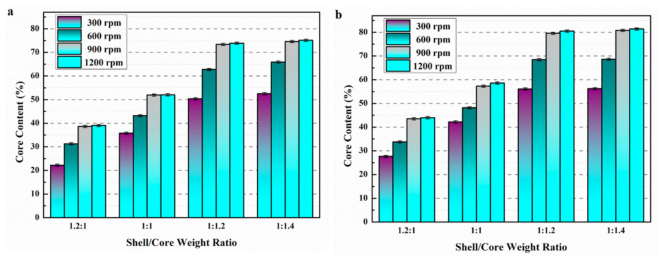

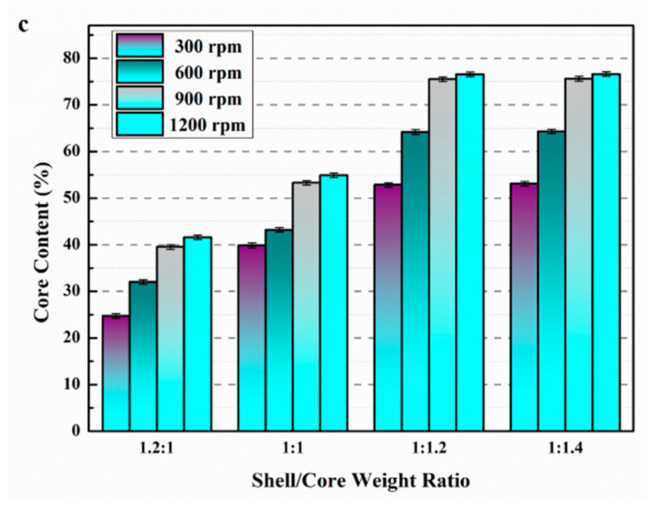
Core content of microcapsules for different preparation parameters. (**a**) 95 °C, (**b**) 105 °C, and (**c**) 115 °C.

Figure 4 represents the core content of microcapsules at different preparation parameters. As can be seen from Figure 4, the core content of microcapsules continued to increase when the shell/core weight ratio was decreased from 1.2:1 to 1:1.2 at the same preparation temperature and stirring speed. When the shell/core weight ratio was decreased from 1:1.2 to 1:1.4, the core content of the microcapsules did not increase significantly. This was due to the limited ability of the microcrystalline wax to encapsulate excess E-51 epoxy resin.

The core content of microcapsules increased significantly while the stirring speed increased from 300 to 900 rpm. When the stirring speed continued to increase from 900 to 1200 rpm, there was no obvious change in the core content of microcapsules. This may be due to the poor dispersion and adhesion of E-51 epoxy resin at low stirring speed, and the microcrystalline wax could not encapsulate E-51 epoxy resin well, resulting in the low core content of microcapsules. However, with the increase of stirring speed, the microcrystalline wax/E-51 epoxy resin mixture was evenly dispersed, and the core content of microcapsules increased significantly.

Compared with Figure 4a–c, the core content of microcapsules increased accordingly when the preparation temperature was elevated from 95 to 105 °C. However, the core content of the microcapsules decreased when the preparation temperature was increased from 105 to 115 °C. This is due to the rapid quenching and solidification of the core material by the coated shell material to prepare the microcapsules. When the preparation temperature was lower (95 °C), the viscosity of the liquid microcrystalline wax increased and the shell of the microcapsules became thicker, which eventually led to a decrease in the core content of the microcapsules [27]. However, when the preparation temperature reached 115 °C, the perfluorotributylamine could not allow the microcrystalline wax to cool, solidify, and encapsulate E-51 epoxy resin rapidly due to the high temperature, which led to the decrease of microcapsule core content. The core content of microcapsules was higher (74.61%) when the shell/core weight ratio was 1:1.2, the stirring speed was 900 rpm, and the preparation temperature was 105 °C.

### 3.2. Particle Size Distributions

**Figure 5:**
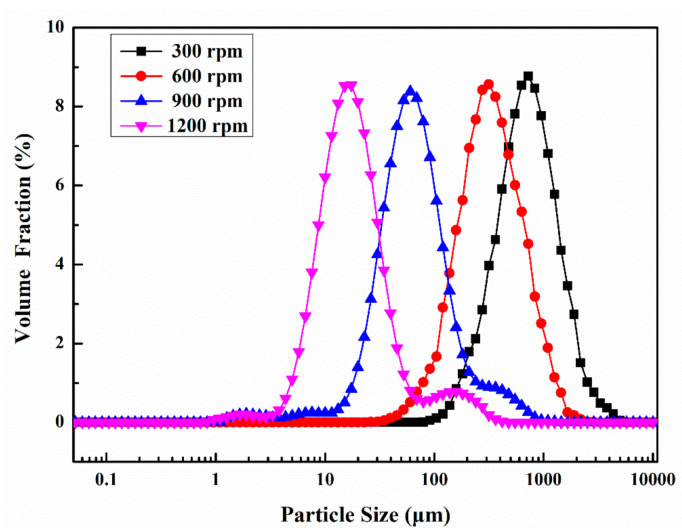
Particle size distribution of microcapsules at different stirring speeds.

Figure 5 demonstrates the particle size distribution of microcapsules prepared at 105 °C with a shell/core weight ratio of 1:1.2 and at different stirring speeds. The D10, D50, and D90 values of the microcapsules are shown in Table 3. In Figure 5, the particle size distribution of microcapsules indicated a decreasing trend with increasing stirring speed. As shown in Table 3, when the stirring speed was 300 rpm, the particle size distribution of microcapsules was 316–1200 μm. After the stirring speed reached 600 rpm, the particle size distribution of microcapsules was 104–831 μm. For the microcapsules with stirring speed of 900 rpm during the preparation process, the particle size distribution declined significantly to only 23–182 μm. The particle size distribution of microcapsules varied from 7 to 52 μm when the stirring speed went up to 1200 rpm. According to Table 3, the average particle sizes of microcapsules prepared at 300, 600, 900, and 1200 rpm were 630, 275, 60, and 17 μm, respectively. This is because during the preparation of microcapsules, with the increase of stirring speed, the magnetic stirring bar provided higher shear force to the microcrystalline wax/E-51 epoxy resin mixture. When the stirring speed is higher, the liquid microcrystalline wax is dispersed more uniformly under the high shear force, and the E-51 epoxy resin will be dispersed in microcrystalline wax in the form of smaller droplets. Therefore, when perfluorotributylamine is added, microcapsules with smaller particle size are likely to be generated during the solidification process.

### 3.3. Thermal Properties Analysis

**Figure 6:**
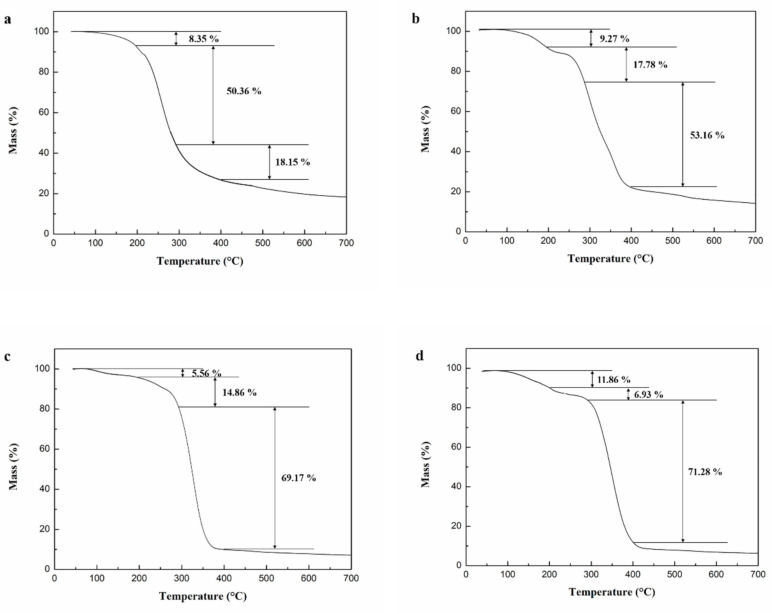
TG curves of microcapsules prepared with different shell/core weight ratios. (**a**) 1.2:1, (**b**) 1:1, (**c**) 1:1.2, and (**d**) 1:1.4.

The thermogravimetric (TG) curves of the microcapsules prepared with different shell/core weight ratios are illustrated in Figure 6. The preparation temperature and stirring speed of the microcapsules were 105 °C and 900 rpm, respectively. Figure 6 indicates that the mass loss of microcapsules was mainly distributed in three temperature ranges: 100–198, 198–280, and 280–396 °C. Among them, the mass loss in the range of 100–198 and 198–280 °C was caused by the melting and decomposition of microcrystalline wax. However, the protective effect of the microcapsule shell on the microcapsule core material was weakened due to the destruction of the shell material. The mass loss of microcapsules in the range of 280–396 °C was caused by the volatilization of N, N-dimethylformamide, and the thermal decomposition of epoxy resin.

As shown in Figure 6a, when the shell/core weight ratio of microcapsules was 1.2:1, the mass of microcapsules decreased by 8.35%, 50.36%, and 18.15% in the range of 100–198, 198–280, and 280–396 °C, respectively. However, the mass of microcapsules decreased by 9.27%, 17.78%, and 53.16% in the ranges of 100–198, 198–280, and 280–396 °C, respectively, while the shell/core weight ratio of microcapsules declined to 1:1 (Figure 6b). In Figure 6c,d, the mass loss of microcapsules increased to 69.17% and 71.28% in the range of 280–396 °C as the shell/core weight ratio continued to drop to 1:1.2 and 1:1.4. The results indicated that the mass loss of microcapsules increased significantly with the decrease of shell/core weight ratio at temperatures from 280–396 °C, indicating that more E-51 epoxy resin was encapsulated into the microcrystalline wax. This is consistent with the results in Section 3.1.

Taking into account the influence of preparation temperature, stirring speed, and shell/core weight ratio on the core content, particle size distribution, and thermal properties of the microcapsules, the optimal process parameters for the preparation of microcrystalline wax coated E-51 epoxy resin microcapsules were microcrystalline wax/E-51 epoxy resin weight ratio of 1:1.2, stirring speed of 900 rpm, and preparation temperature of 105 °C.

### 3.4. Morphology of Microcapsules

**Figure 7:**
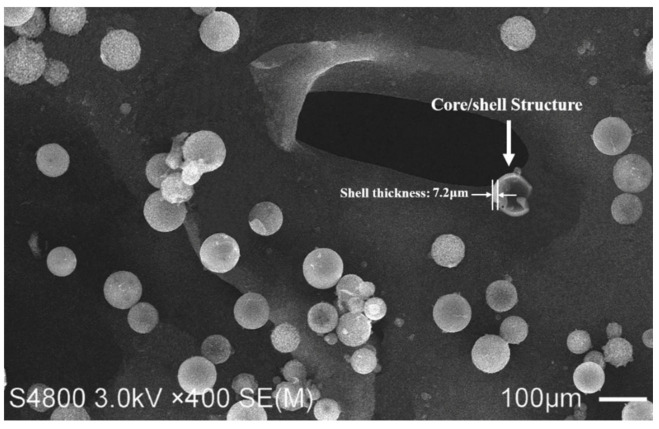
Morphology of microcapsules.

The morphology of intact and ruptured microcapsules prepared with a microcrystalline wax/E-51 epoxy resin weight ratio of 1:1.2 and a stirring speed of 900 rpm at 105 °C is shown in Figure 7. As seen from the SEM image, the microcapsules have a regular spherical shape and a rough surface, which facilitates good adhesion between the matrix of the cementitious material and the shell of the microcapsules. The particle size distribution of the microcapsules varied from 50 to 80 μm, which was consistent with the test results in Section 3.2. The SEM image also indicated the shell thickness of a ruptured microcapsule with a diameter of 60 μm was 7.2 μm, which suggested that the shell thickness of the microcapsule was about 1/9 of its diameter. The results indicated that microcapsules prepared under optimal process parameters with a high diameter/shell thickness ratio, which could provide a larger storage capacity for the encapsulation of E-51 epoxy resin.

### 3.5. FTIR Analysis

**Figure 8:**
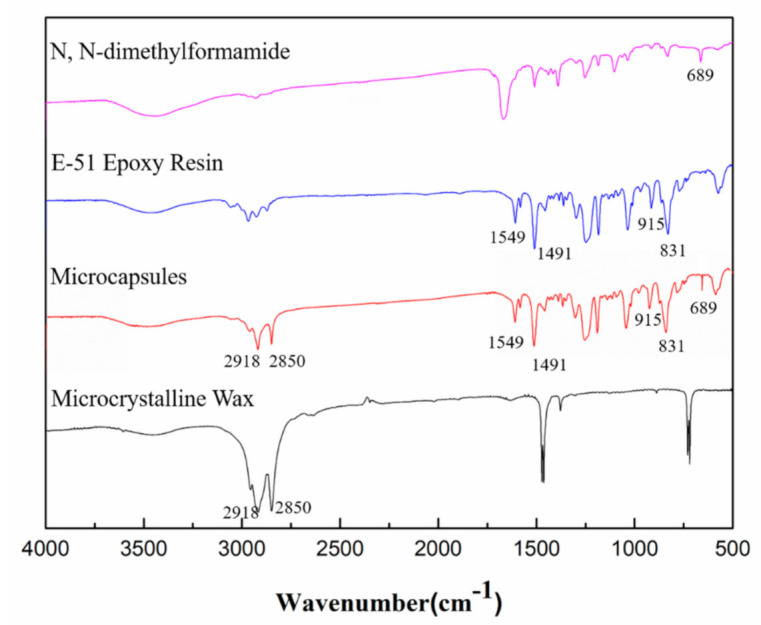
FTIR spectra of microcrystalline wax, microcapsules, E-51 epoxy resin, and N, N-dimethylformamide.

Figure 8 describes the FTIR spectra of microcrystalline wax, microcapsules, E-51 epoxy resin, and N, N-dimethylformamide. The microcapsules were prepared under optimal process parameters. It can be noticed that the peaks at 2918 and 2850 cm^−1^ in Figure 8 are symmetric and asymmetric stretching vibrations of -CH_2_- and -CH_3_ groups of microcrystalline waxes. The absorption peaks at 1549 and 1491 cm^−1^ are mainly due to the stretching vibrations of aromatic C–C and symmetric bending absorption peaks of dimethyl in epoxy resin. The absorption peaks at 915 and 831 cm^−1^ are characteristic absorption peaks of C–O and C–O–C in the ethylene oxirane group. In addition, the characteristic peak of the amide I band was found at 689 cm^−1^, indicating that the microcapsules contain a small amount of N, N-dimethylformamide. The SEM photo in Section 3.4 shows that the microcapsules have a core/shell structure with a large space inside to encapsulate the healing agent. The combined SEM, TG, and FTIR results indicate that E-51 epoxy resin was successfully encapsulated in the microcrystalline wax.

### 3.6. Mechanical Properties

The preparation parameters of microcapsules used in the self-healing test were the weight ratio of microcrystalline wax/E-51 epoxy resin was 1:1.2, the stirring speed was 900 rpm, and the temperature was 105 °C.

**Figure 9:**
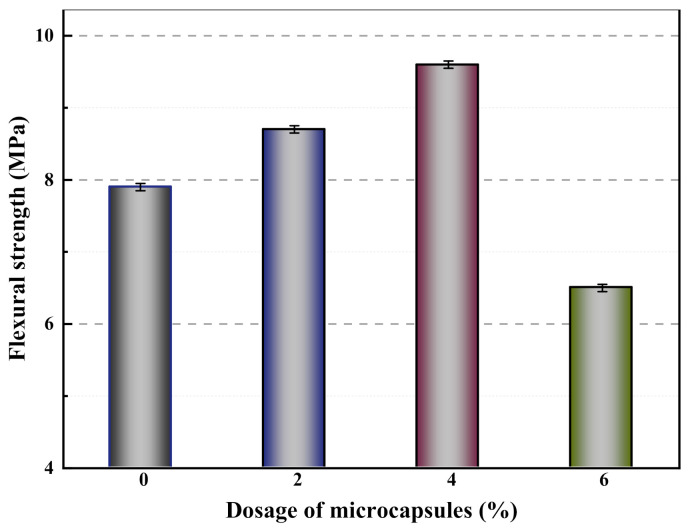
Flexural strength of mortars containing microcapsules.

Figure 9 shows the flexural strength of mortars containing different contents of microcapsules after 28 days of standard curing. From Figure 9, the flexural strengths of M-0 (control mortar), M-1 (mortar with 2% cement weight microcapsules), M-2 (mortar with 4% cement weight microcapsules), and M-3 (mortar with 6% cement weight microcapsules) are 7.9, 8.7, 9.6, and 6.5 MPa, respectively. Compared with M-0, the flexural strength of M-1 and M-2 increased by 10.1% and 21.5%, respectively. When the microcapsule content increases to 6% (M-3), the flexural strength of mortar is reduced by 17.7% compared with M-0. The results showed that the mechanical properties of the mortar were enhanced and then decreased with the increase of microcapsule content. This is due to the large difference in Young’s modulus between the microcapsules and the cement matrix, and the addition of microcapsules causes gaps in the bonding surface of the microcapsules and the matrix material. The more microcapsules content, the more pores in the matrix material, and consequently, the stiffness is reduced and the flexural strength also declined. However, the addition of a small dosage of microcapsules is conducive to improving the fluidity and compactness of the mortar, making the structure of the mortar compact and improving the flexural strength [34].

**Figure 10:**
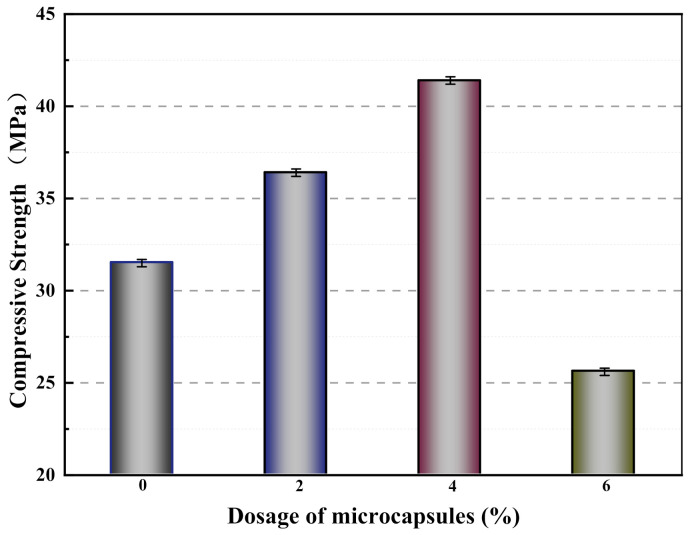
Compressive strength of mortars containing microcapsules.

The compressive strengths of mortars containing different microcapsule contents after 28 days of standard curing are indicated in Figure 10. The compressive strengths of M-0, M-1, M-2, and M-3 were 31.5, 36.4, 41.4, and 25.6 MPa, respectively. Compared with M-0, the compressive strengths of M-1 and M-2 increased by 15.6% and 31.4%, respectively. The reason for this is that mortar is a mixture of materials with different particle sizes, and there is a certain dosage of voids in the mortar. A suitable dosage of the microcapsules can fill the internal voids of the mortar and improve its compressive strength [27]. In contrast, the compressive strength of M-3 decreased by 5.7% compared with that of M-0. This may be due to the large difference in modulus between the microcapsules and the cement matrix, and when excessive many microcapsules are mixed into the mortar, the bonding surface of the microcapsules and the matrix material is prone to gaps. The increase of these gaps both reduces the compactness of the mortar in the molding process and affects the particle gradation of the cementitious material, which eventually leads to the decrease of the compressive strength of the mortar.

### 3.7. Compressive Strength Recovery Ratio

**Figure 11:**
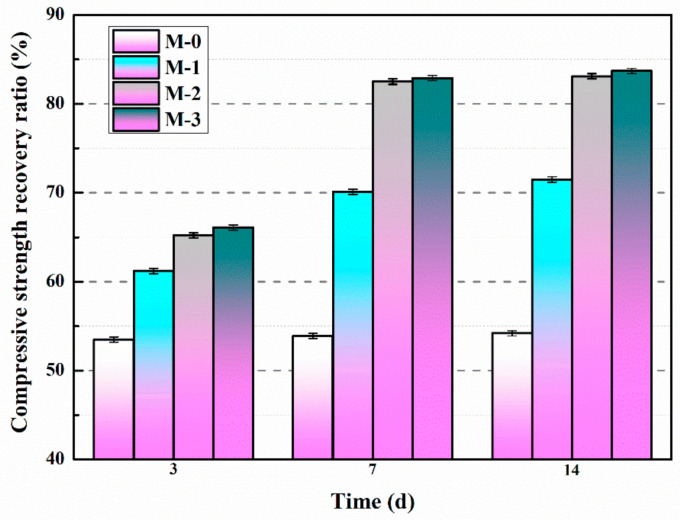
Compressive strength recovery ratios of mortars containing microcapsules.

Figure 11 describes the compressive strength recovery ratios of mortars containing different contents of microcapsules with variable self-healing time. According to Figure 11, the compressive strength recovery ratio of M-0 does not change with increasing self-healing time, indicating that the intrinsic self-healing ability of cementitious materials cannot effectively self-heal the pre-damaged M-0 within 14 days.

**Figure 12:**
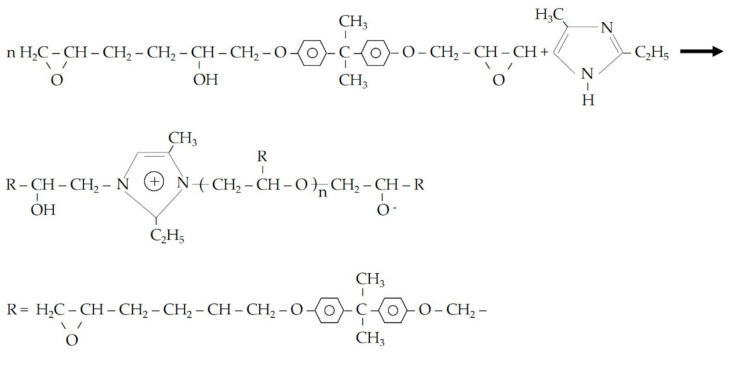
Chemical reaction process between E-51 epoxy resin and 2-ethyl-4-methylimidazole.

Compared with M-0, the compressive strength recovery ratios of M-1, M-2, and M-3 after self-healing were significantly higher, as shown in Figure 11. The trends of the compressive strength recovery ratios of M-1, M-2, and M-3 were all improved rapidly from day 3 to day 7, and slightly changed from day 7 to day 14, indicating that the compressive strength of the pre-damaged mortar recovered after 14 days of self-healing. This is mainly due to the appearance of microcracks in the mortar after preloading, and the stress at the crack tip would rupture the shell of the microcapsules. The E-51 epoxy resin flows into the microcracks and reacts with 2-ethyl-4-methylimidazole (pre-mixed in the mortar) to cure, and the reaction products eventually make the microcracks self-healing. The chemical reaction process between E-51 epoxy resin and 2-ethyl-4-methylimidazole is described in Figure 12.

After 14 days of self-healing, the compressive strength recovery ratios of M-0, M-1, M-2, and M-3 were 54.2%, 71.5%, 83.1%, and 83.7%, respectively. Compared with M-0, the compressive strength recovery ratios of M-1, M-2, and M-3 increased by 31.9%, 53.3%, and 54.4%, respectively. The results showed that the compressive strength recovery ratios of the mortar increased with the increase of microcapsule content. This is mainly due to the fact that the higher the microcapsule content, the more E-51 epoxy resin can produce the curing reaction, which leads to the increase of compressive strength recovery ratio. When the microcapsule content exceeds 4%, the compressive strength recovery ratio of mortar does not change much, which indicates that the self-healing limit of the microcapsules to the internal microcracks of the mortar has been reached.

### 3.8. Pore Size Distribution

For cementitious materials, it is generally considered that pores with a diameter of less than 0.1 μm are harmless or, at least, less harmful, and pores with a diameter of more than 0.1 μm are harmful and have a strong influence on the mechanical properties and impermeability of cementitious materials [28]. In this section, the pores with a particle size greater than 0.1 μm are referred to as harmful pores.

**Figure 13:**
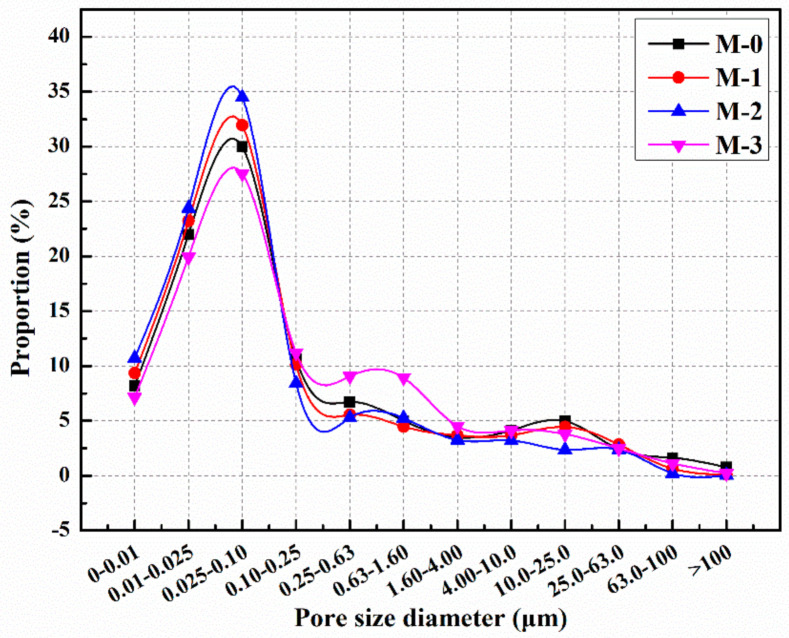
Pore size distribution of cement mortars with microcapsules.

Figure 13 shows the pore size distribution of mortars with different microcapsule contents after standard curing for 28 days. From Figure 13, it can be seen that the proportion of harmful pores of M-0, M-1, M-2, and M-3 are 39.8%, 35.5%, 30.4%, and 45.4%, respectively. Compared with M-0, the proportion of harmful pores in M-1 and M-2 decreased by 10.8% and 23.6%, respectively. The results showed that the proportion of harmful pores of cement mortar decreased as the microcapsule content increased from 0% to 4%, indicating that the compactness of cement mortar improved. This is because cement mortar is a mixture of various materials with different particle sizes. If an appropriate dosage of microcapsules is added during the preparation process, the particle gradation of the mortar can be optimized, the internal structure of the mortar can be improved, and the compactness of mortar can be increased, thus reducing the proportion of harmful pores in mortar. The proportion of harmful pores of M-3 in Figure 13 is 14.7% higher than that of M-0, which indicates that with the increase of the content of microcapsules, the bonding interface between microcapsules and cement matrix easily produces gaps, which reduces the compactness of mortar and leads to the increase of the proportion of harmful pores in mortar.

**Figure 14:**
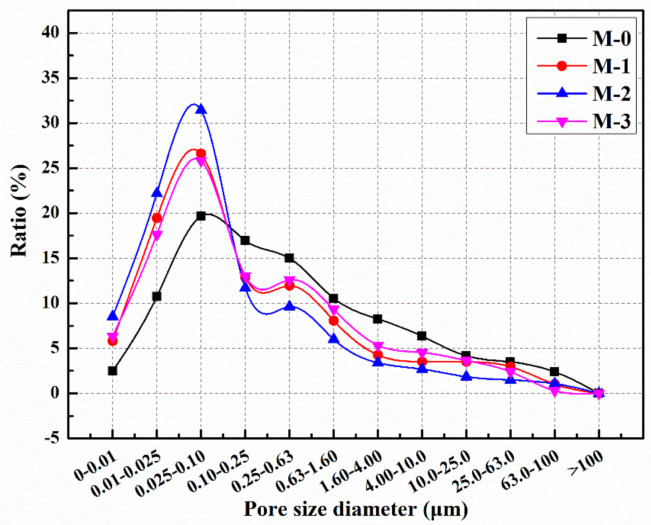
Pore size distribution of cement mortar with microcapsules after self-healing for 14 days.

After 14 days of self-healing, the pore size distribution of the pre-damaged mortar containing different contents of microcapsules is shown in Figure 14, and the proportions of harmful pores in M-0, M-1, M-2, and M-3 were 67.1%, 48.1%, 37.8%, and 51.2%, respectively. The results indicate that the proportion of harmful pores of the mortar containing microcapsules decreased significantly after self-healing. The reason is that microcracks appear inside the mortar after preloading, and the stress at the tip of the cracks will destroy the shell of the microcapsules. E-51 epoxy resin flows out from the microcapsules and cures with 2-ethyl-4-methylimidazole in the mortar to fill the microcracks, refine the pore size, improve the compactness of the mortar, and reduce the proportion of harmful pores.

### 3.9. Chloride Diffusion Coefficient Recovery Rate

**Figure 15:**
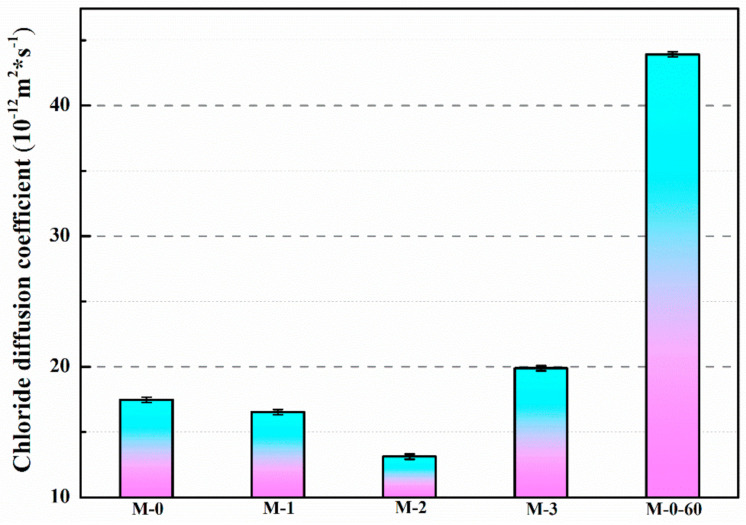
Chloride diffusion coefficient of mortar containing microcapsules. (M-0-60: the control mortar after 60% fb0 pre-load.)

Figure 15 represents the chloride diffusion coefficients of mortars containing different contents of microcapsules after 28 days of standard curing. The chloride diffusion coefficients of M-0, M-1, M-2, M-3, and M-0-60 were 17.48 × 10^−12^, 16.53 × 10^−12^, 13.12 × 10^−12^, 19.88 × 10^−12^, and 43.91 × 10^−12^ m^2^/s, respectively. Compared with M-0, the chloride diffusion coefficients of M-1 and M-2 were reduced by 5.4% and 24.9%, respectively. When the addition of microcapsules was increased to 6% (M-3), the chloride diffusion coefficient of mortar increased by 13.7% compared with M-0. This is due to the fact that after adding the appropriate dosage of microcapsules in the preparation process, the internal structure of mortar is improved and the compactness rises because of the filling effect of microcapsules on mortar, which improves the impermeability of mortar and reduces the chloride diffusion coefficient of mortar. However, with the increase of contents of microcapsules, the bonding interface between the microcapsules and cement matrix tends to produce gaps, and these gaps provide channels for the flow and transmission of chloride ions, leading to the rise of the chloride diffusion coefficient, which decreases the impermeability of mortar.

**Figure 16:**
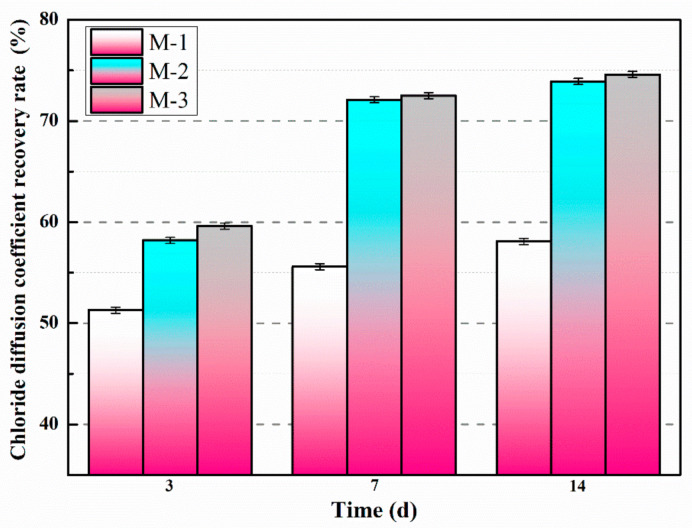
Chloride diffusion coefficient recovery rates of mortars containing microcapsules.

The chloride diffusion coefficient recovery rates of the mortars with microcapsules at different self-healing time are described in Figure 16. The chloride diffusion coefficient recovery rates of M-1, M-2, and M-3 after self-healing were significantly improved from Figure 16. The chloride diffusion coefficient recovery rates of M-1, M-2, and M-3 increased rapidly from day 3 to day 7, and remained essentially constant from day 7 to day 14. After 14 days of self-healing, the chloride diffusion coefficient recovery rates for M-1, M-2, and M-3 were 58.1%, 73.9%, and 74.6%, respectively. This is mainly due to the presence of microcracks in the mortar after preloading, and the stress at the tips of the microcracks can rupture the microcapsule. The released E-51 epoxy resin cured in contact with 2-ethyl-4-methylimidazole and self-healed the microcracks. As the dosage of microcapsules in the mortar rises, the content of E-51 epoxy resin available for self-healing also increases, thereby improving the self-healing ability of the mortar containing microcapsules.

### 3.10. Ultrasonic Test Analysis

**Figure 17:**
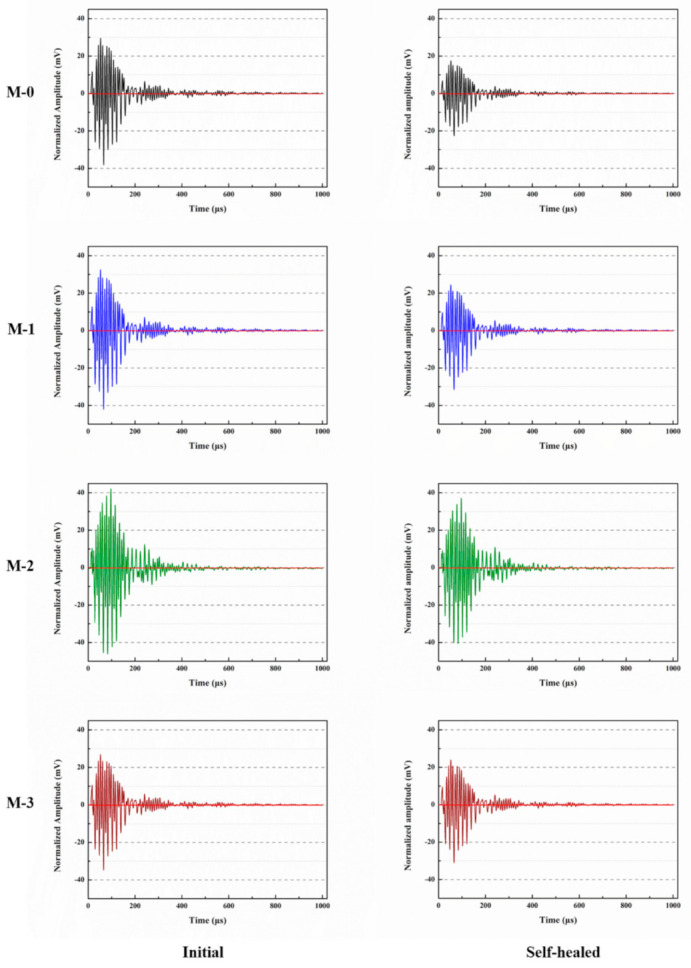
Ultrasonic waveform of mortars with microcapsules before and after self-healing.

Figure 17 shows the ultrasonic waveforms before and after self-healing of mortars with different contents of microcapsules. It can be observed from Figure 17 that the maximum amplitudes of M-0, M-1, M-2, and M-3 were 29.63, 32.59, 42.15, and 26.93 mV, respectively, after 28 days of standard curing. Compared with M-0, the maximum amplitudes of M-1 and M-2 increased to a certain extent, while the maximum amplitude of M-3 declined. This is because when the ultrasonic waves propagate in mortar, the acoustic impedance of mortar is much larger than that of air, so when pores are encountered, the ultrasonic signal attenuates, resulting in a decrease in the amplitude of the received signal [39]. When the compactness of the mortar is higher, the ultrasonic signal encounters fewer internal defects such as pores, the signal attenuation amplitude is smaller, and the wave amplitude is relatively larger. According to Section 3.8, M-2 has a lower harmful pores proportion, so its amplitude is higher.

Figure 17 also illustrates that the maximum amplitudes of M-0, M-1, M-2, and M-3 were 17.51, 24.44, 37.09, and 23.97 mV, respectively, after 14 days of self-healing. The results indicate that the maximum amplitude of mortars containing microcapsules recovered significantly after 14 days of self-healing. After the mortar was pre-damaged, microcracks and pores appeared inside the mortar, which caused the ultrasonic signal to attenuate and the maximum amplitude to reduce. However, under the stress of the crack tip, the microcapsules rupture and release E-51 epoxy resin. E-51 epoxy resin can solidify with 2-ethyl-4-methylimidazole and fill in the microcracks and pores, so that the compactness of the mortar rises and the maximum amplitude is recovered.

**Figure 18:**
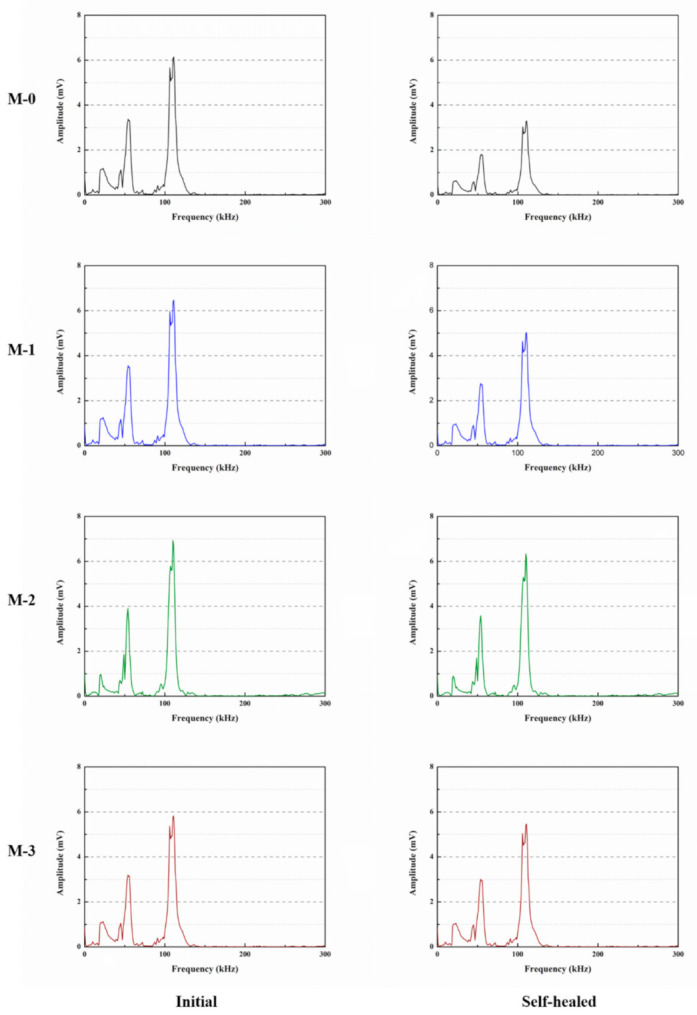
Ultrasonic frequency of mortars with microcapsules before and after self-healing.

The Fast Fourier Transform (FFT) of the Gaussian function can estimate the variation of the ultrasonic waves and obtain clearer results [40]. The ultrasonic shapes of M-0, M-1, M-2, and M-3 in Figure 17 were FFT transformed to investigate their frequency domain curves before and after self-healing. Figure 18 shows the ultrasonic frequencies of the mortars with microcapsules before and after self-healing. For the mortars after 28 days of standard curing, the dominant frequencies of the test were around the resonance frequency 107 kHz. The peak in the low frequency range is due to the air in the pores of the mortar, which can attenuate the ultrasonic energy [40]. The maximum amplitudes of the dominant frequencies were 6.14, 6.47, 6.93, and 5.82 mV for M-0, M-1, M-2, and M-3, respectively. The main reason is that the addition of the appropriate content of microcapsules reduces the internal pores of the mortar and increases the compactness, so the maximum amplitude of the dominant frequency was increased. However, after 14 days of self-healing, the maximum amplitudes of the dominant frequencies of M-0, M-1, M-2, and M-3 were 3.17, 5.03, 6.34, and 5.46 mV, respectively. The results displayed the dominant frequencies of M-1, M-2, and M-3 recovered obviously, and the FFT analysis proved that the mortar with 4% cement weight of microcapsules had better self-healing ability.

Considering the influence of the content of microcapsules on the mechanical properties, impermeability, pore size distribution, ultrasonic testing, and self-healing ability of mortars, the optimal content of microcapsules in mortar is 4% of the weight of cement.

### 3.11. Surface Cracks Self-Healing

**Figure 19:**
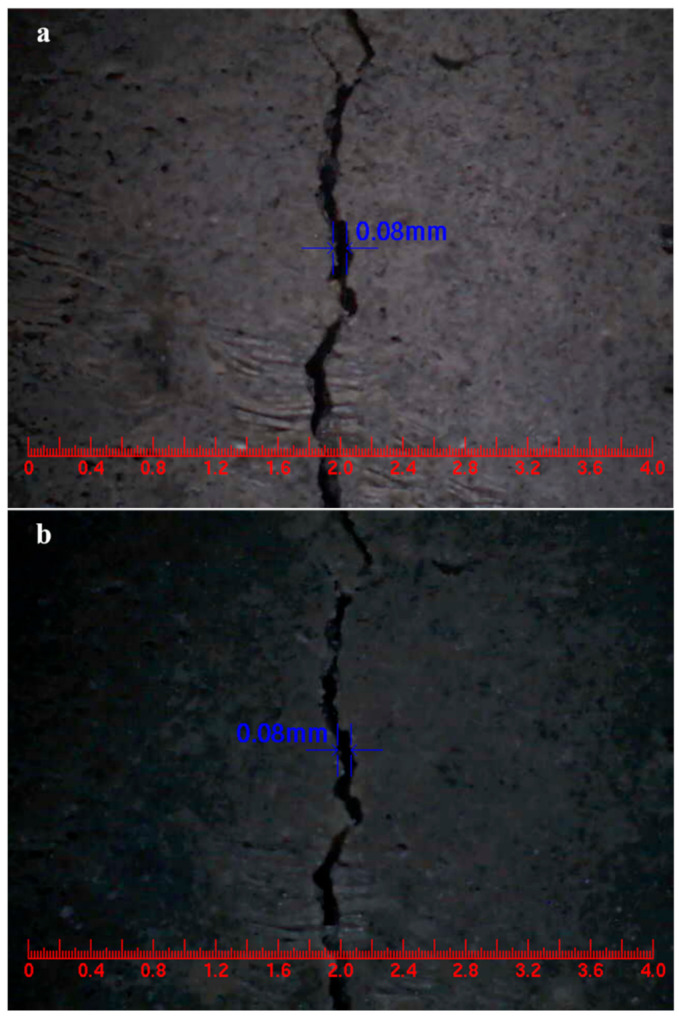

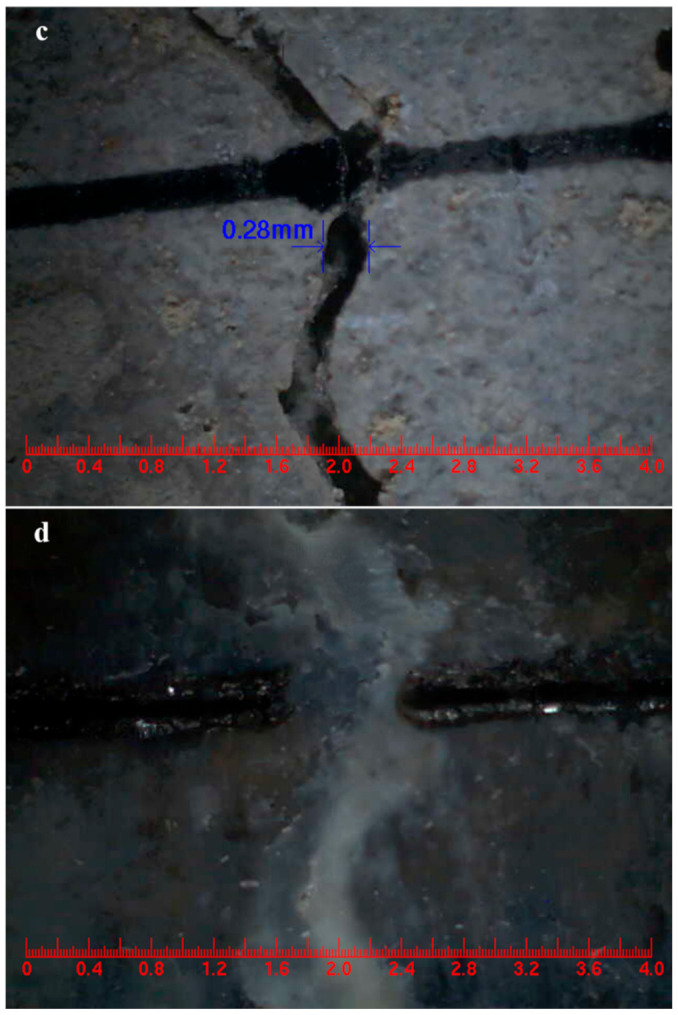
Self-healing of surface cracks in mortar. (**a**) Control mortar, (**b**) control mortar after three days of self-healing, (**c**) mortar containing microcapsules, (**d**) mortar containing microcapsules after three days of self-healing.

The purpose of developing self-healing cementitious materials is to obtain good healing properties when cracks occur in cementitious materials. Therefore, the main objective of this study was to provide convincing and direct evidence for the self-healing ability of cementitious materials containing microcapsules. To achieve this objective, mortar specimens with microcapsules were pre-cracked by the three-point bending method, and then the healing of the cracks was measured. The content of microcapsules in the mortar was 4% of the cement weight. Comparing Figure 19a,b, the initial width of the surface crack in the control mortar was 0.08 mm, and the width of the surface crack did not change after three days of self-healing. However, it could be found from Figure 19c,d that the surface cracks with an initial width of 0.28 mm in the mortar containing microcapsules were self-healing after three days. The results indicate that the mortar containing microcapsules could rapidly self-heal surface cracks with widths less than 0.28 mm.

## 4. Conclusions

Microcrystalline wax containing epoxy resin microcapsules for self-healing of cementitious materials was prepared using the melt–dispersion–condensation method in this paper. The core content, particle size distribution, thermal properties, morphology, and chemical structure of the microcapsules were characterized. The self-healing ability of mortars containing different contents of microcapsules was also evaluated. The main conclusions are as follows:

(1) The optimal parameters of microcapsules were microcrystalline wax/E-51 epoxy resin weight ratio of 1:1.2, stirring speed of 900 rpm, and preparation temperature of 105 °C. The core content, average particle size, and mass loss (280–396 °C) of the microcapsules prepared with the optimal parameters were 74.61%, 60 μm, and 69.17%, respectively. SEM and FTIR results could prove that E-51 epoxy resin was successfully encapsulated in the microcrystalline wax, and the diameter/shell thickness ratio of the microcapsules was about 9/1.

(2) When the content of microcapsules was 0% to 4% of the weight of cement, the mechanical properties, impermeability, and maximum amplitude of mortars were improved, and the harmful pores proportion decreased. However, when the content of microcapsules continued to rise to 6% of the weight of cement, the mechanical properties, impermeability, and maximum amplitude of mortars declined, and the harmful pores proportion raised. The optimal content of microcapsules in mortar is 4% of the cement weight.

(3) After 14 days of self-healing, the compressive strength recovery ratio, harmful pores proportion, chloride diffusion coefficient recovery rate, maximum amplitude, and dominant frequency of M-2 were 83.1%, 37.8%, 73.9%, 37.09 mV, and 6.34 mV, respectively. Surface cracks with an initial width of 0.28 mm in the mortar containing microcapsules were self-healed after three days. The results indicate that microcapsules can rapidly self-heal surface cracks in mortar.

## Figures and Tables

**Table 1:** Chemical composition of cement (%).

Chemical Composition	SiO_2_	Al_2_O_3_	CaO	MgO	SO_3_	Fe_2_O_3_	Loss
Portland Cement	22.34	6.29	58.92	2.08	2.32	3.44	2.12

**Table 2:** Mortar formulations by mass ratio.

Mortars	Cement	Sand	Water	Microcapsules
M-0	100	50	300	0
M-1	100	50	300	2
M-2	100	50	300	4
M-3	100	50	300	6

**Table 3:** Particle size of microcapsules with varying stirring speeds.

Stirring Speeds/rpm	D10 Values/μm	D50 Values/μm	D90 Values/μm
300	316	630	1200
600	104	275	831
900	23	60	182
1200	7	17	52

D10 value indicates 10% of the microcapsules with a volume diameter less than this value, D50 value indicates 50% of the microcapsules with a volume diameter less than this value, and D90 value indicates 90% of the microcapsules with a volume diameter less than this value.

## Data Availability

Data sharing not applicable.

## Supplementary Materials

The following are available online at https://www.mdpi.com/article/10.3390/ma14071725/s1, Size distribution of microcapsules; pore size distribution of mortar.

## Abbreviations

M-0	Control mortar
M-1	Mortar with 2% of cement mass microcapsules
M-2	Mortar with 4% of cement mass microcapsules
M-3	Mortar with 6% of cement mass microcapsules
M-0-60	Control mortar after 60%f_b0_ preloading
FFT	Fast Fourier Transform
