# Supplementary material for: Preparation and Characterization of Microcrystalline Wax/Epoxy Resin Microcapsules for Self-Healing of Cementitious Materials
Source: Materials (Basel). 2021 Mar 31;14(7):1725. doi: 10.3390/ma14071725 (PMC8037595; doi:10.3390/ma14071725)
Supplement: Supplementary file 1 [file materials-14-01725-s001.pdf]

materials

Supplementary Materials

Preparation and Characterization of Microcrystalline Wax/Epoxy Resin Microcapsules for Self-Healing of Cementitious Materials

Wei Du [1,2,*] Quantao Liu [2], Runsheng Lin [3], Xin Su [1,*]

1 School of Material Science and Chemical Engineering, Ningbo University, Ningbo 315211, China
2 State Key Laboratory of Silicate Materials for Architectures, Wuhan University of Technology, Wuhan 430070, China; liuqt@whut.edu.cn
3 College of Engineering, Department of Architectural Engineering, Kangwon National University, Chuncheon-si 200-701, Korea; linrunsheng@kangwon.ac.kr
* Correspondence: duwei@nbu.edu.cn (W.D.); suxin@nbu.edu.cn (X.S.)

Citation: Du, W.; Liu, Q.; Lin, R.; Su, X. Preparation and Characterization of Microcrystalline Wax/Epoxy Resin Microcapsules for Self-Healing of Cementitious Materials. *Materials* **2021**, *14*, 1725. https://doi.org/10.3390/ma14071725

Academic Editor: Theodore E. Matikas

Received: 3 March 2021
Accepted: 30 March 2021
Published: 31 March 2021

Publisher's Note: MDPI stays neutral with regard to jurisdictional claims in published maps and institutional affiliations.

Copyright: © 2021 by the authors. Licensee MDPI, Basel, Switzerland. This article is an open access article distributed under the terms and conditions of the Creative Commons Attribution (CC BY) license (http://creativecommons.org/licenses/by/4.0/).

Size Distribution of Microcapsules:

The average size and size distribution of synthesized microcapsules produced under different process parameters were determined using a laser particle size analyzer (Mastersizer 2000, Malvern Instruments Ltd., Malvern, UK). Before the test, microcapsules were placed in a drying box at 40 °C for 24 h. Then, 1 g microcapsules were dispersed by 50 mL deionized water in the analysis box. During the laser diffraction measurement, particles were passed through a focused laser beam. These particles scatter light at an angle that was inversely proportional to their sizes. The angular intensity of the scattered light was measured via a series of photosensitive detectors. The size distribution of the after-sieving microcapsules was determined from the OM images of microcapsules using a commercial dimensional measurement software.

Pore Size Distribution of Mortar:

The nuclear magnetic resonance (NMR) spectrometer (MesoMR25) was used to measure pore size distribution of the mortars. The measuring principle of pore size distribution by NMR technique is that the water in the pore will resonate with the molecule under low power magnetic field, relaxation occurs during the process of energy exchange and release and the relaxation time can reflect the pore size. NMR is suitable for measuring pore size distribution of 2 nm–1 mm, which has been applied to the study of pore structure of cement-based materials. The size of mortar sample used for NMR test was 25 mm × 25 mm × 15 mm. Prior to the test, the mortar sample was wiped to clean the surface powder and was vacuum-saturated with water for 24 hours and then stored in water for another week. The resonance frequency was 23.40 MHz, temperature of the magnet was 32.00 ± 0.02 °C and instrument probe diameter was 25 mm.
